# Mechanisms of “Cough-Trick” for Pain Relief during Venipuncture: An Experimental Crossover Investigation in Healthy Volunteers

**DOI:** 10.1155/2019/9459103

**Published:** 2019-12-12

**Authors:** Taras I. Usichenko, Henriette Janner, Maria Gagarine, Dragan Pavlovic, Eric Lang, Klaus Hahnenkamp

**Affiliations:** ^1^Department of Anesthesiology, University of Greifswald, Greifswald, Germany; ^2^Department of Anesthesia, McMaster University, Hamilton, Canada; ^3^University of Ottawa, Ottawa, Canada; ^4^Department of Anesthesia, Dalhousie University, Halifax, Canada

## Abstract

**Objectives:**

The easily performed “cough-trick” (CT) reduces pain during venipuncture (VP), although the underlying mechanism remains unclear. The aim was to investigate the pain-reducing effect of CT during VP in comparison with two distraction methods, as well as under the influence of naloxone.

**Methods:**

54 healthy male volunteers participated in 3 investigations. Pain during standardized VP with CT was compared to a “weak” distraction (squeezing a rubber ball; investigation 1; *n*=20) and to a “strong” distraction (inflating a tourniquet to a given pressure; investigation 2; *n*=21). In investigation 3 (*n*=13), pain at a VP without intervention was compared to pain at VP with CT under naloxone; pressure pain thresholds before and after naloxone administration were also measured. Pain was assessed using a 100 mm visual analogue scale. Data were compared within each sample using Student's *t*-test for paired samples.

**Results:**

Pain intensity at VP with CT was lower than under “weak” distraction (mean difference 5 mm; 95% CI: 0.5 to 9.6; *P*=0.03). Pain levels under CT and “strong” distraction were comparable. There was no difference between pain under CT after naloxone infusion and pain without intervention. Pressure pain threshold decreased (mean difference 1 mm; 95% CI: 0.1 to 1.0 mm; *P*=0.02) after naloxone administration.

**Conclusion:**

Pain-reducing effect of CT during VP is superior to that of simple motor distraction and equivalent to a complex distraction method. This might be due to the activation of segmental pain inhibitory pathways during coughing indicated through the lack of pain reduction due to CT under opioid antagonist blockage.

## 1. Introduction

“Cough-trick” (CT)—coughing on command simultaneously to skin puncture—is a simple effective method of pain relief during peripheral venipuncture (VP) and various injections [1–3]. Several investigations confirmed the effectiveness of CT either with a classic technique [1–3] or in modified form as various breathing interventions (e.g., Valsalva maneuver or balloon inflation) in pain relief during peripheral VP, vaccination injections, spinal puncture, and even biopsy [1–9]. Classic CT is easily performed in adult [1, 2] and pediatric populations [3, 4] without requiring additional equipment, costs, or efforts by the staff like other conventional methods for pain reduction during VP, e.g., applying or injecting local anesthetics, performing the Valsalva maneuver, or using balloon inflation [4–14].

Whereas the underlying mechanisms of the pain reduction effects of CT still remain unclear, several ideas were proposed to explain this phenomenon. CT could be a distraction at the moment of VP, which is a well-known method of pain reduction through the shift of attention to a nonnoxious stimulus in the immediate environment [15]. However, the results of studies on pain relief through distraction are controversial [16–18]. Another explanation could be the activation of the segmental pain inhibitory pathways due to the increased pressure in the subarachnoid space during coughing mediated by vagal afferents [19–21].

In order to study the underlying mechanism of the pain-reducing effect of CT at peripheral VP, we conducted a double-blinded crossover study consisting of three investigations to compare the pain relief effect of CT. We compared pain during VP accompanied by (1) “weak distraction” in one sample; (2) “strong distraction” in a second sample, and (3) the effect of CT under the block of opioid receptors using naloxone, with pain during VP without any intervention in the third sample of volunteers.

We hypothesized that CT would have greater pain-reducing effects than the “weak” and “strong” distraction methods since we speculated that the underlying mechanisms of CT are based on the activation of the endogenous opioid system rather than on distraction. Furthermore, we tested whether the hypoalgesic effects of CT disappear after the administration of the nonselective antagonist of opioid receptors (naloxone).

## 2. Methods

### 2.1. Study Design and Participants

This prospective experimental study consisted of three double-blinded controlled crossover investigations in three different samples and was approved by the local ethics committee of the University Medicine of Greifswald. Written informed consent was obtained from all participants. Students of the University of Greifswald were recruited via announcement according to the following eligibility criteria: healthy male volunteers, aged 20 to 40 years, a physical status I according to American Society of Anesthesiologists (ASA) classification as well as with a straight run of the vein on the dorsum of the nondominant hand at least 4 cm long and 3 mm thick. Exclusion and discontinuation criteria were as follows: intake of analgesics, antidepressant and anticonvulsant medication, anticoagulants and/or antiplatelet agents, sedatives or alcohol, a history of peripheral neuropathy, abnormal skin conditions (infection, scars, psoriasis, and eczema) at the site of the VP, unsuccessful VP, and an inflamed site of VP within 1 week after VP. All volunteers were instructed to take a light breakfast at least 30 minutes prior to VP, since hypoglycemia was found to accompany various pain conditions, induce hyperalgesia, and aggravate pain [22]. All sessions were conducted in the same laboratory room between 07:00 and 10:00 a.m. in order to minimize the influence of circadian differences in pain sensitivity [23].

### 2.2. Investigation 1 and Investigation 2

To test the hypoalgesic effects of CT in comparison with the two different distraction methods, two investigations were conducted in two different samples of healthy male subjects whereas the study procedure only differed in the distraction method used as comparing condition. In both investigations, participants visited the study room for the first time at least 3 days prior to the sessions with VP, to diminish situational anxiety. For blinding purposes, participants were informed that the aim of the study would be to compare the intensity of pain during VP with two different sizes of a new kind of indwelling venous catheters. During this first visit, the ear lobule was punctured with an Ascensia Blood Sugar Meter (Bayer Vital GmbH, Leverkusen, Germany) to measure the initial serum glucose concentration, and meanwhile, subjects were acquainted with the pain scoring procedure on a 100 mm visual analogue scale (VAS-100).

Participants were randomly allocated to two groups depending on whether the VP would be performed at first with the CT and then three weeks later in the control condition or the other way around. The order of conditions was randomized by flipping a coin. The intersession interval was chosen to prevent carry-over effects.

**Figure 1:**
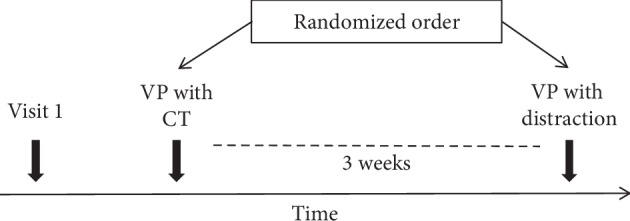
Study design of investigation 1 and investigation 2. Visit 1 was performed at least 3 days prior to the first session with venipuncture (VP) to acquaint subjects to the environment. During the following three weeks, participants performed two sessions in a randomized order with a VP that was accompanied by either a cough-trick (CT) or a distraction method.

In the first session with VP, participants were placed in the supine position and a tourniquet was fastened on the nondominant arm to ensure that the vein on the dorsum of the hand, chosen for VP, became clearly visible. The same vein on the nondominant hand of each subject was punctured twice within three weeks by the same investigator (“puncturer”), who has had experience with peripheral VP (Figure 1). VP was always performed with 20 G “Safelon” cannula (BD Medical, Heidelberg, Germany).

#### 2.2.1. Cough-Trick Procedure

CT procedure was performed as described previously [1]. In brief, after the vein had become clearly visible on the dorsum of the hand, the subjects were asked to turn their head to the opposite side of the VP. Then, they were instructed to perform a single cough with a moderate intensity without moving their arms, as a test cough. Immediately after, the volunteers were asked to cough again. VP was performed simultaneously to this second cough [1].

#### 2.2.2. Distraction Procedures

As “weak” distraction (investigation 1), volunteers took a rubber ball in the dominant hand and were instructed to squeeze it simultaneously to the VP.

As “strong” distraction (investigation 2), participants were instructed to inflate the tourniquet, on the arm on which VP was performed, to a pressure of 200 mmHg and then to actively hold this pressure at 200 mmHg during the VP (for about 3 seconds).

### 2.3. Investigation 3

Investigation 3 was an exploratory study performed to examine whether the hypoalgesic effects of CT disappear after the administration of nonselective antagonist of opioid receptor naloxone. The subjects of the third sample were invited to one session and were told that the aim of the study was to examine whether injection of naloxone would influence the perceived pain during VP. Participants were instructed that they would get either a naloxone or saline solution and they would be blinded regarding their group allocation. At the beginning of the session, a VP, as described for investigations 1 and 2, without CT intervention was performed in the dominant hand of each participant. Immediately after, a naloxone infusion 0.075 mg/kg (CuraMed Pharma GmbH, Karlsruhe, Germany) was given via this intravenous access within 10 minutes by one investigator (“puncturer”), which is with the half-life of 30–80 min.

**Figure 2:**
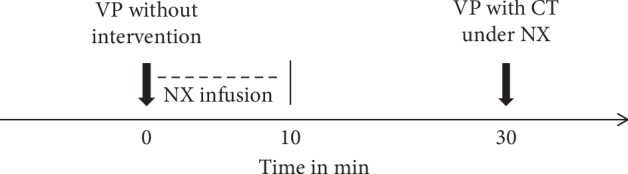
Study design of investigation 3. Venipuncture (VP) without intervention was performed prior to the administration of nonselective opioid receptor antagonist naloxone (NX infusion). 30 minutes later, a second VP with a cough-trick (CT) under naloxone impact was performed. Pain intensity was assessed at both VPs and compared.

Twenty minutes following the end of naloxone infusion, another VP on the nondominant hand was performed and participants were instructed to do the CT at this VP as described above (Figure 2).

### 2.4. Outcome Measures

In all investigations, pain intensity during VP was assessed as ratings on a 100 mm visual analogue scale (VAS-100) ranging from 0 (no pain) to 100 (worst pain imaginable) as primary outcome. For this purpose, a second investigator (“assessor”), who was asked to leave the room during the VPs in order to be blinded regarding the interventional conditions, showed the VAS-100 scale to the participant immediately after VP was performed. In investigation 3, pressure pain thresholds before and after naloxone injection were also measured using a mechanical pressure algometer (0 to 8 points on a 3 cm scale) applied at the glabella whereas the pressure was increased until the participants reported instead of pressure the first sensation of pain.

**Figure 3:**
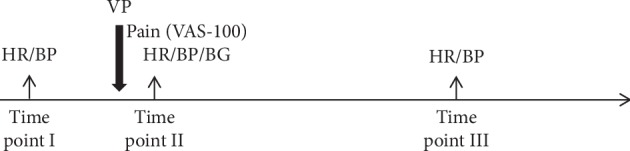
Time points of outcome measurement. Time point (I): measurement of heart rate (HR) and blood pressure (BP) 5 minutes before the venipuncture (VP). Time point II: assessment of pain intensity at VP on a 100 mm visual analogue scale (VAS-100); incidence of hand withdrawal; and HR, BP, and blood glucose (BG) directly after the VP. Time point III: measurement of heart rate and blood pressure 10 minutes after the VP.

As secondary outcomes, serum glucose concentration was recorded after each VP. Heart rate (using pulse oximetry) and blood pressure (using oscillometry) were displayed and recorded on an IntelliVue MP70 monitor (Philips, Amsterdam, the Netherlands) taken on the alternate arm 5 minutes before (time point I), immediately after (time point II), and 10 minutes following the VP (time point III; Figure 3). The effectiveness of blinding was verified after the last session by asking the volunteers if they had any doubts about the proclaimed aim of the study and whether they recognized the cough-trick as a method for pain reduction during VP.

### 2.5. Statistical Analysis

To test for differences between pain ratings, heart rate, blood pressure, and serum glucose concentration within each sample, the data were compared using Student's *t*-test (for normally distributed data) or Wilcoxon signed-rank test (for nonparametric data) for paired samples. Mean differences (MD) with 95% CI were given as treatment effect estimates, where appropriate. Two-sided *P* values <0.05 were regarded as statistically significant.

Statistical analysis was conducted using the Statistical Package for Social Science version 22.0 (SPSS, IBM Corporation, New York, USA). Data are presented as mean ± standard deviation.

The sample size calculation for investigations 1 and 2 was based on data of our previous study [1] with a two-sided level of significance of 5% and a power of 90%. Expecting a difference of at least 25% between the mean pain intensity during VP with CT and both distraction procedures and using the value of standard deviation of 16 mm from the previous investigation, the sample estimation resulted in a size of 19 subjects per group/condition to assure statistically significant results for investigation 1 and investigation 2 of the study. Investigation 3 was an exploratory investigation, and thus, we have estimated the sample size of 15 volunteers on the basis of previous studies with comparable methodology [24, 25].

## 3. Results

Venipuncture was successful on the first attempt in all study participants.

### 3.1. Investigation 1 and Investigation 2

Twenty-one male participants, aged 24 ± 3 (mean ± standard deviation) years, were included in investigation 1 and 20 male volunteers, aged 26 ± 4 years, were enrolled in investigation 2 according to the eligibility criteria. The characteristics of the samples for each investigation of the study are displayed in Table 1.

**Figure 4:**
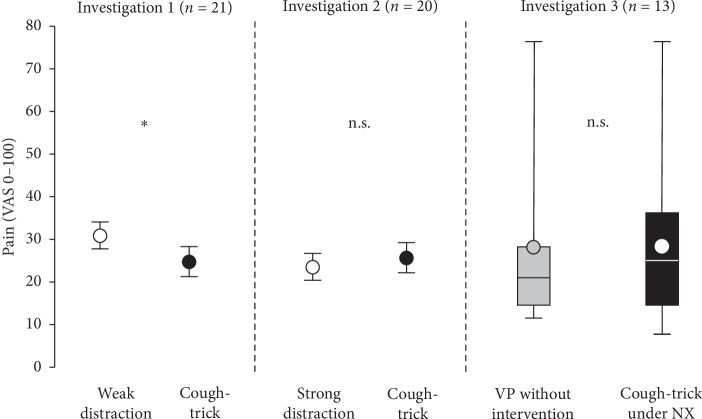
Pain intensity during all conditions and investigations. Pain intensities at venipuncture (VP) were assessed on a 100 mm visual analogue scale (VAS-100). Normally distributed data from investigations 1 and 2 are presented as mean and standard error of the mean. Nonparametric data from investigation 3 median are presented as median, interquartile range, and minimum and maximum values for each condition; circles within the boxes are the mean values of pain intensity. Cough-trick (CT) was performed simultaneously to the VP. Weak distraction: participants squeezed a rubber ball with the nonpunctured hand during the VP procedure. Strong distraction: participants manually inflated the tourniquet, placed on the arm at which VP was performed, to a pressure of 200 mmHg and hold it attentively during VP. Without intervention: VP was performed without pain relief CT intervention prior to the administration of nonselective opioid receptor antagonist naloxone (NX). Cough-trick under NX: VP with CT was performed 30 minutes after naloxone infusion. ^*∗*^*P*=0.03 for the comparison of cough-trick vs. weak distraction with Student's *t*-test for paired samples.

In investigation 1, pain intensity during VP was lower under CT (27 ± 13 mm) than under “weak” distraction (32 ± 15 mm; mean difference (MD) = 5 mm; 95% CI: 0.5 to 9.6; *P*=0.03) (Figure 4).

In investigation 2, pain intensities during VP under CT (28 ± 15 mm) and under “strong” distraction (25 ± 14 mm; MD = 3 mm; 95% CI: −8.0 to 2.7; *P*=0.3) did not differ substantially (Figure 4).

There were no significant differences among the secondary outcomes between the conditions in both investigations.

Less than 4 participants reported retrospective doubts about the proclaimed aim of the study in each investigation, which indicates successful blinding in both investigations (Table 2).

### 3.2. Investigation 3

For the third sample, 15 male subjects were recruited. One participant was excluded due to a vasovagal reaction at the first VP without CT. Another participant did not perform the CT properly during VP. Therefore, data of 13 subjects, aged 26 ± 4 years, were available for final analysis.

Pain under CT 30 min after naloxone injection was 26 (22) mm (as given here as median (interquartile range)) and did not differ from pain during VP without intervention 23 (13) mm before the naloxone injection (*P*=0.8; Wilcoxon signed-rank test; Figure 4).

Pressure pain threshold after naloxone injection (7 ± 2 mm) decreased if compared to pain threshold before naloxone injection (6 ± 2 mm; MD = 1 mm; 95% CI: 0.1 to 1.0; *P*=0.02). Blood glucose level increased after naloxone administration in comparison with the level before naloxone injection (5.2 ± 0.8 vs. 4.8 ± 0.9 mmol/l; MD = 0.4; 95% CI: 0.1 to 0.6; *P*=0.004). The hemodynamic parameters before, during, and after naloxone injection were comparable.

Since only three participants reported doubts about the proclaimed aim of the study, blinding can be regarded as successful (Table 2).

## 4. Discussion

In this prospective randomized controlled crossover study, we examined the underlying mechanisms of the cough-trick (CT) as pain relief intervention during peripheral venipuncture (VP) in healthy volunteers. Thereby, we compared the effects of CT with two different kinds of distraction methods that differed in their complexity regarding the cognitive and motor efforts required by both procedures. Furthermore, we investigated the potential involvement of the endogenous opioid system in the pain relief effect of CT by administering a nonselective opioid receptor antagonist naloxone.

We have found that CT was more effective than squeezing of a rubber ball during VP as a simple motoric task used as a “weak” distraction method for pain relief. This is in line with findings on the superiority of the Valsalva maneuver and balloon inflation regarding pain reduction during VP if compared to “weak” distraction of squeezing the rubber ball [5, 7].

However, no differences in pain during VP were revealed when comparing CT to a “strong” distraction method where participants should manually inflate the tourniquet placed on their arm and keep the pressure on a certain level during the VP procedure. Both distraction strategies differed in the amount of motor as well as cognitive efforts that were necessary to fulfill the tasks. Manual inflation of the tourniquet with continuous pressure control requires a stronger shift of attention towards the nonnoxious stimulus as well as more cognitive efforts if compared to the squeezing of a rubber ball. Furthermore, there is a greater competing sensory input due to the pressure of the tourniquet in comparison with the simple motor task. The findings support the hypothesis that greater distraction is associated with greater reduction in perceived pain [15]. The individual analgesic response to distraction procedures might be influenced by the attention of the participant to distractor [26]. The low variability of response to distraction in participants from investigations 1 and 2 (according to comparable standard deviations of pain intensity mean values during all VP procedures with analgesic interventions) can be explained by good ability of young subjects to concentrate themselves on simple external distraction cues.

No hypoalgesic effect of CT was found 30 minutes after the infusion of naloxone, even though pressure pain threshold was decreased and blood glucose levels increased, indicating increased pain sensitivity and metabolic stress reaction after the administration of naloxone. The lack of expected pain reduction due to CT under the block of opioid receptor antagonist points towards the involvement of the endogenous opioid system in the pain relief effect of CT. The increased pressure in the subarachnoid space during coughing may have activated baroreceptors due to the compression of vessels, which resulted in the activation of the segmental pain inhibitory pathways mediated by vagal afferents [19–21].

The low level of pain intensity on VP without CT among the participants from investigation 3 (median 23 mm) in comparison with the previous literature on that topic (where the reported mean values exceeded 35 mm on a 100 mm VAS) [1, 3, 5, 6] can be explained due to accidental prevalence of individuals, who reported lower pain intensity on VP. Out of 13 volunteers included, 10 reported pain intensity less than 30 on a 100 mm VAS. However, we believe that this finding (relatively low pain perception of VP without CT in volunteers from investigation 3) did not influence the result of the investigation, since the participants demonstrated consistently low levels of pain perception throughout investigation, which yielded reliable comparison result in a crossover design.

Nevertheless, the interpretation of this data may be limited due to the small sample size used to investigate the impact of naloxone on CT. Moreover, CT under naloxone was not compared to the effects of CT under a placebo (saline) injection in a randomized crossover manner. Generally, the major limitations of the study are the small sample sizes and that only male subjects were examined. The effectiveness of CT should be examined under various circumstances and in a large population of male and female subjects of different ages and ethnicities, since, for example, Wallace et al. observed differences of ethnicity regarding the effectiveness of the CT procedure in their study [3].

The findings in our study clearly point towards the superiority of the CT procedure over other methods for pain reduction during VP. CT is more effective than a simple motor distraction and as effective as an even more complicated cognitive-motoric task. Mutlu and Balcı found both balloon inflation and CT are comparable regarding their effectiveness in reducing pain during VP [4]. Schmid et al. and Bogani et al. reported that CT reduced pain as much as the injection of local anesthetics during cervical and colposcopy-guided biopsy [8, 9]. Also, Valsalva maneuver was found to reduce pain during VP as effectively as the application of the eutectic mixture of local anesthetics [27]. In a recent study of Yilmaz and Güneş, where Valsalva maneuver, rubber ball squeezing, and CT were examined regarding their hypoalgesic effects during peripheral intravenous catheterization, CT was found to exert the strongest reduction in pain [28]. In contrast to other nonpharmacological methods, CT does not require any additional equipment or expertise and even younger children and people that would not be able to perform the described distraction methods due to disabilities and/or impairments could probably perform the CT easily [5].

## 5. Conclusion

Findings from our study demonstrate that CT, without requiring any additional equipment, is more effective than a comparably simple motoric distraction task in reducing pain during venipuncture and equivalent to a more complicated distraction method. The pain relief effect of CT is probably based on the activation of segmental pain inhibitory pathways during coughing indicated through the lack of pain reduction during CT under opioid receptor blockade.

## Figures and Tables

**Table 1:** Sample characteristics.

	Investigation 1	Investigation 2	Investigation 3
Conditions	Cough-trick^a^ vs. weak distraction^b^	Cough-trick vs. strong distraction^c^	Without intervention^d^ vs. cough-trick under NX^e^
Sample size (*n*)	21	20	13
Age in years	24 ± 3	26 ± 4	26 ± 4
Height in cm	180 ± 6	181 ± 8	181 ± 4
Weight in kg	77 ± 9	74 ± 11	81 ± 9

^a^ *Cough-trick*: moderate cough performed simultaneously to the venipuncture (VP). ^b^*Weak distraction*: participants squeezed a rubber ball with the nonpunctured hand during VP. ^c^*Strong distraction*: participants manually inflated the tourniquet, placed on the arm at which VP was performed, to a pressure of 200 mmHg and hold it attentively during VP. ^d^*Without intervention*: VP was performed without intervention to inject naloxone. ^*e*^*Cough-trick under NX*: cough performed during VP under naloxone (NX) impact. Data are presented as mean ± standard deviation.

**Table 2:** Number of subjects reporting doubts about the proclaimed aim of the study.

		Investigation 1_*a*_	Investigation 2_*a*_	Investigation 3_*b*_
Doubts about study aim?	No	18	16	10
	Yes	3	4	3

^*a*^Proclaimed aim of the study was to compare the pain intensity during venipuncture with two different sizes of a new kind of indwelling venous catheters. ^*b*^Proclaimed aim of the study was to examine whether injection of naloxone would influence perceived pain during VP.

## Data Availability

The datasets collected during the current study are available from the corresponding author.
